# CFD estimation of gas production in tight carbonates using single and dual-porosity models

**DOI:** 10.1038/s41598-023-48450-5

**Published:** 2023-12-19

**Authors:** Syed Oubee Khadri, Ibnelwaleed A. Hussein, Fadhil Sadooni, Ezeddin Shirif

**Affiliations:** 1 Gas Processing Center, Qatar University, P.O. Box 2713, Doha, Qatar; 2 Department of Chemical Engineering, Qatar University, P.O. Box 2713, Doha, Qatar; 3 Environmental Science Center, Qatar University, P. O. Box 2713, Doha, Qatar; 4 Program of Petroleum Systems Engineering, University of Regina, Regina, SK S4S 0A2 Canada

**Keywords:** Engineering, Chemical engineering

## Abstract

Tight Carbonate reservoirs are regarded as one of the most complex reservoir formations due to the heterogeneity and complexity of their mineral composition, pore structure, and storage model. It is uncommon to study the implementation of a transport model appropriate for such formation. Recent studies focused on tight reservoirs and developed models for shale or coal bed methane reservoirs. This study proposes a single and dual-porosity transport model that solely considers the tight matrix and acidized region to shed light on the transport models for tight carbonates. The numerical model included the effect of transport mechanisms such as Knudsen diffusion, desorption, and viscous flow. The proposed transport model includes the apparent permeability model defining these transport mechanisms. Finite element method analysis was conducted on the numerical model using COMSOL Multiphysics. Due to the presence of nanopores in both shale and tight Carbonate, transport models proposed for the former can be utilized to determine the fluid flow behavior in the latter. The adsorption isotherm, rock density, pore structure, porosity, and permeability of the tight carbonate reservoir, which contrasted with the shale results, were the defining features of the reservoir used in the transport model. The dual-porosity model yielded a peak production of 104,000 m^3^/day, whereas the proposed model represents a shallow production rate from the single-porosity reservoir. The results were validated with an analytical solution proposed in the literature. Based on the literature findings and the production profile, the desorption did not play a significant role in the total production due to calcite’s low affinity towards CH_4_.

## Introduction

Tight carbonate reservoirs are among the most complex due to their vast heterogeneity. Most affirmed petrophysical parameters undergo some degree of alteration over time due to diagenesis, unconformities, impurities or other materials, and environmental factors. These variables affect the reservoir's porosity and permeability and, consequently, the modeling of the fluid flow in the reservoir. According to Rashid et al.^1^, nearly half of all global oil reserves are estimated to be stored in carbonate rocks. Since carbonate is the most common form of the reservoir in the Middle East, which contributes and has already contributed about 15% of the world's hydrocarbon reserves, these reservoirs can be easily inferred. The study of tight carbonate is challenging due to diagenetic processes, which modify the pore's microstructure. These processes are compaction, dissolution, Precipitation, stylolitisation, cementation, and fracturing. Each process occurring during rock formation influences a morphological change in the rock microstructure.

The objective of production analysis is crucial in determining the hydrocarbon reservoir's feasibility. Different studies are conducted to develop reservoir models and determine production profiles from these reservoirs. Shale and other unconventional reservoirs tend to be complex due to the deviation from the conventional approach. Shale reservoirs generally are formations with porosity in nanoporous segments, which would be challenging to utilize conventional means of analysis. Thus, the methodology utilized for unconventional reservoirs such as tight carbonate, shale, and coal bed methane is exceedingly complex.

The standard Darcy rule is unsuitable to represent the flow in this type of deposit due to nanopores in tight carbonate reservoirs. According to Li et al.^2^, non-Darcy and stress sensitivity effects are crucial for evaluating the well productivity of tight carbonate gas reservoirs. In contrast to inertial effects, this study focuses on non-Darcy flow with changing permeability due to growing stress sensitivity. Including apparent permeability into the continuity model can make it easier to develop a representative transport model, which is more straightforward and versatile in defining different production models to be implemented in developing these reservoirs.

According to Radwan et al.^3^, the pore networks, while crucial for hydrocarbon reservoirs, are vastly affected due to changes over time. The complexity of the fracture distribution's "vast" permeability, diagenetic history, and carbonate deposition features utilized to develop a petrophysical carbonate model is challenging. To address this challenge, integrated and multiscale datasets are needed to analyze these types of reservoirs. However, integrating the pore network into production analysis increases computational time.

Previous studies on ultimate gas recovery provided different approaches to determining a representative transport model for tight reservoirs. However, these models used a specific flow mechanism rather than combining multi-models. Furthermore, studies on tight carbonate reserves are generalized as conventional reservoirs^4^, whereas they include intricate nano-pore systems^5–7^. Such systems do not conform to Darcy Law due to the diversity in pore structure; hence, the flow mechanisms, such as Knudsen Diffusion occurrence^8,9^. Generally, the gas transport models can be compiled as Navier–Stokes Equation or Darcy Law utilizing apparent gas permeability for the reserve permeability. Therefore, applying the gas apparent permeability model to the Navier–Stokes Equation would provide the required transport model. Minimal studies have determined a representative gas transport model for Tight Carbonate Reservoirs.

This paper uses single and dual-porosity models to estimate ultimate gas production (UGR) in tight carbonates. The models used in this study are based on tight shale and coal bed methane models because these formations generally have similar porous media. However, specific flow mechanisms in these nanopores have different contributions to storage and transport; hence, some modifications are implemented in the gas apparent permeability corrections. Based on the literature, tight carbonates have negligible adsorptive/desorptive properties during exposure to methane; this aspect will be accounted for in the modified proposed models utilized to estimate UGR. In addition, the model is parametrically evaluated depending on how permeability, porosity, and wellbore pressure affect ultimate gas recovery. This work addresses whether tight carbonates and shales have similar flow mechanisms where a dual continuum and transport model could be used for both formations.

## Methodology

### Characteristics of tight carbonate formations and similarities with tight shale formations

Understanding the geological characteristics of Tight Carbonate formations would lay the groundwork for developing a representative production analysis model. Petrophysical data such as porosity, permeability, and relative permeability is required in any reservoir simulation. George et al.^10^ compiled the critical aspects of developing tight carbonate reservoir models. These aspects included challenges and strategies for developing field production strategies for these formations. Tight carbonate reservoirs are intricate due to their higher heterogeneity. These evaluations were conducted on field data from Abu Dhabi, UAE. The average permeability of the formation ranged from 0.5 to 5 mD, which is relatively higher than shale or other tight formations. The formation typically consists of discrete fractures, adversely increasing permeability.

Rashid et al.^1,6^ studied different petrofacies in tight carbonate formations. The study classified the Kometan formation into three different lithological units. Petrofacies A is characterized by compacted and cemented formation, which provides a poor reservoir quality with porosity ranging from 0.005 to 0.1 and permeability ranging from 65 nD to 51 µd. However, with natural fractures, the permeability adversely increases to 9.75 mD, improving the reservoir quality. Petrofacies B refers to dissolved packstone with vuggy pores, comprising porosity ranging from 0.2 to 0.3 and permeability ranging from 0.087 mD to 4.1 mD (Refer Figs. 11 and 12 from^6^). Finally, Petrofacies C, determined to be mudstone carbonate, has undergone dolomitization and has poor reservoir quality relative to Petrofacies B, which was determined by its permeability ranging from 0.065 to 5 mD. By inferring the analysis done by this study, it is safe to assume that tight carbonate formation generally comprises vugs, pores, and fractures, contributing to the reservoir's final permeability and productivity index^6^. Similar findings were determined in the Jiuquan Basin formation in China^11^.

Li et al.^12^ characterized a tight carbonate reservoir using a modified physics model. The study compiled potential pore structures to define a suitable rock physics model. Based on log data, lab measurements, and core analysis, the permeability contributors are vugs, pores, and fractures. The study also laid a foundation for the complexity of the said formation due to its heterogeneity. However, the type of formations is divided during any characterization between vuggy, tight, and fractured formations^13^. Tight carbonate rocks are defined as carbonate formations with less than 2% porosity and permeability of less than 0.1 mD^14^.

Regarding adsorptive properties, tight carbonate reservoirs generally have less affinity toward the common gases present in the reservoir, such as CH_4_. Nevertheless, the formation has more affinity toward CO_2_, which can hint toward its storage potential for CO_2_^5^. Elbashier et al.^15^ studied the geomechanical effects of gas adsorption in tight Carbonate. In contrast to the adverse geomechanical effects of gas transport in shales, calcite depicts a lower strain during the physisorption of CH_4_ molecules, concluding that the transport in tight carbonate does not adversely affect any geomechanical changes irrespective of pressure or density^15^. Based on the results above, it can hint at the viability of its storage potential for CO_2_^5^.

Kong et al.^11^ comprehensively characterized tight carbonate rock from the Jiuquan Basin. The formation consists mainly of oil while also possessing properties of ‘tight’ formation properties. The pore structure characteristics were studied to understand oil transport in nanopores. The transport in nanopores does not always conform to the continuum mechanics; therefore, it is vital to determine the transport mechanism present in this volume. The study provided typical characteristics of tight carbonate reservoirs in general. The principal characteristic was the pore throat which was determined to be in the range of 0.01–0.1 and 1–10 µm, in which the latter pore size distribution holds most of the pores present in the matrix^11^.

Freitag et al.^13^ conducted an extensive petrophysical characterization of tight carbonates of the Upper Jurassic. SEM imaging (Refer Fig. 5 from^13^) shows that the pore structure in tight carbonates comprises both nanopores and micropores^5,7^. The permeability is contributed mainly due to fracture permeability, which interconnects pores. Shale and tight Carbonate are similar while dealing with transport mechanisms due to similar pore structure, complexity, and heterogeneity. However, compared to other tight gas reservoirs, tight carbonate exhibits a more significant heterogeneity^6,16,17^.

### Apparent permeability and transport models of tight reservoirs

Gas apparent permeability models can be used to define the different flow mechanisms in the nanoporous media. Considering Fongmaxi shale core, Chen et al.^18^ investigated non-linear gas transport under several thermal conditions. The study utilized numerical models incorporating the temperature effects into the final ultimate recovery. The analysis was done on shale samples which included flow mechanisms such as slip flow, bulk diffusion, Knudsen diffusion, and adsorption–desorption. The temperature adversely affected the final ultimate gas recovery from the shale core. The resulting trend follows the power law model at the operating temperatures; the production increased with the increase in temperature. The study did not include a specific numerical solution unique to cores; instead, it utilized gas flow in the shale reservoir model. Gas extraction is an endothermic process, so studying the temperature impact on the final production is essential^18^.

1 $$Q=S\left(r\rho \frac{{k}_{o}}{\mu }\frac{\partial p}{\partial r}+r{D}_{e}\frac{\partial }{\partial r}\left(\frac{pM}{zRT}\right)\right)=S(r\frac{pM}{zRT}{A}{\prime}{\left(\frac{\partial p}{\partial \xi }\right)}^{n+1}{\left(\frac{\partial \xi }{\partial r}\right)}^{n+1}+r\frac{M}{zRT}A{\left(\frac{\partial p}{\partial \xi }\right)}^{m+1}{\left(\frac{\partial \xi }{\partial r}\right)}^{m+1})$$

Hosseini et al.^19^ utilized gas evolution data from core samples to determine gas content in shale-gas reservoirs. The study proposed a mathematical model to determine gas volume from the nano-porous core. The model incorporates gas expansion and desorption while determining the original gas. It was developed by first matching gas production data to field data to determine average core pressure. By utilizing the output from the latter, free and sorbed gas is determined at the reservoir condition. The study concluded that the gas content at reservoir conditions determined by analyzing the core samples is much higher than the determination made by traditional analysis. The model incorporated the mass continuity equation with adsorbed gas determination. The resulting governing equation under dimensionless terms was obtained using the Laplace domain. Finally, the proposed model was developed and simulated using CMG–GEM and was confirmed by field data. The study provided a methodology to determine the recovery and reserve from a tight rock formation. According to the study, the variables in such determination were only the adsorbed gas which contributed the majority of reserved in coal bed methane and shale gas reservoirs.

Ettehadtavakkol and Jamali^20^ proposed an experimental methodology and workflow for a canister desorption test for studying fluid flow and rock wettability in shale and Coal Bed Methane. The sample that was utilized for the experiment was Marcellus Shale. The results from the canister desorption test concluded that it agreed with an independent field study done on the sample. Furthermore, the study also concluded that the adsorption parameters of the sample contributed crucially to the permeability measurement. Finally, with such results, a new representative analytical model was proposed. The study proposed analytical solutions with both linear and non-linear isotherms. The partial differential equation proposed determines the system's pseudo-pressure, which can be utilized to determine the gas recovery. The cumulative gas release is identified using fractional gas release. The study refers to the fractional gas release ratio of cumulative gas released from the core to the ultimate cumulative gas recovered when the core reaches the equilibrium pressure.

2 $${F}_{D}\left(p\right)= \frac{{m}_{D}\left({p}_{i}\right)-{\overline{m} }_{D}(p)}{{m}_{D}\left({p}_{i}\right)-{m}_{D}({p}_{e})} ; {\overline{m} }_{D}\left(p\right)= \frac{1}{{R}_{a}}{\int }_{0}^{{R}_{a}}{m}_{D}(p)dr$$

Hu et al.^4^ studied and characterized flow through pore-vug-fracture structures in carbonate reservoirs. The study included CT technology to characterize the pore structure, which included studying vug, fractures, and the matrix under different scales. One of the conclusions made by the study was that the pores are unconnected even if the CT resolution reaches 0.5 µm, which indicates that the carbonate matrix is typically tight. Therefore, fractures are the dominant contributor to the permeability of the carbonate matrix. The study also proposed a numerical model for the low porosity highly heterogeneous rock. Simulations of single-phase flow under reservoir conditions were performed utilizing the proposed model. Due to the high heterogeneity of the carbonate formation, vugs, pores, and fractures cause preferential flow and disturbance in the core.

Furthermore, the study also incorporated a two-phase flow in the analytical model. Unlike other unconventional reservoirs, based on the findings of this study, adsorption plays a minor role in the recovery of the gas. The study included the theory of free flow in carbonate reservoirs of large pores and fractures and seepage flow in small-scale pores. The study concludes that the Navier–stokes equation can fully explain the flow mechanism for larger pore sizes, whereas the Darcy equation is utilized for the micro-pore scale^21^*.*

3 $$\phi \frac{\partial p}{\partial t}-\nabla .\left(\frac{k}{\mu }p\nabla p\right)= \frac{Pa}{\rho ga}{Q}_{s}$$

Kang et al.^22^ proposed a lost gas model based on the methane flow mechanism within porous media under isothermal conditions for shale formation. The study recognizes only lateral flow parallel to shale bedding as the permeability in that direction is dominant, using a mass continuity equation coupled with slippage and adsorption. The observations from the proposed model included changes in the release of desorbed gas and produced gas based on varying matrix temperatures. An algorithm was proposed to calculate the volumetric flux and flow rate. The numerical model results were bench-marked with the volumetric approach in varying adsorption conditions. Molecular modeling was utilized to confirm the submissive adsorption of methane gas on calcite surfaces, concluding that dry gas is trapped in the pore space of this shale as free gas. The concept can also be used for formation with calcite indications such as limestone and carbonates^22^*.*

4 $$\frac{\partial }{\partial t}\left[{\rho }_{g}\phi +\left(1-\phi \right)q\right]= \frac{1}{r}\frac{\partial }{\partial r}(r{\rho }_{g}\frac{{k}_{a}}{\mu }\frac{\partial P}{\partial r})$$

Zhao et al.^23^ proposed a methodology to determine tight reservoir cores' axial and transverse permeability using the Canister Degassing test. The methodology utilizes gas production data from the canister degassing test and correlates it with the analytical solution of the continuity equation in anisotropic core samples to determine the axial and transverse permeability. These results were applied to two shale samples and were bench-marked by conducting a pulse pressure decay test. Results from both tests confirm the practicality of the proposed methodology.

Freeman et al.^24^ studied the microscale behavior of tight and shale gas reservoirs. The modeling of shale or any tight gas reservoir is complex and intricate due to various flow mechanisms. Furthermore, the presence of nanopores further complicates the flow as it moves towards free molecular flow rather than the typical continuum flow. The study includes various models for transport through shale and proposes a new model compatible with conventional petroleum engineering reservoir simulators. In contrast to other formations, shale formation has more transport mechanisms, including convective, Knudsen diffusion, surface diffusion, molecular diffusion, liquid diffusion, and configurational diffusion due to low permeability (mode of 54 nd). The study compares Fick’s law and the Dusty-Gas model regarding its utilization for specific formations depending on the permeability. With permeabilities of 10^–12^ m^2^ or more extensive, extended Fick’s law is implemented, while for lower permeabilities, dusty gas models are suitable. The study also utilizes constant Klinkenberg to characterize flow through shale gas reservoirs.

Wang et al.^16^ analyzed gas flow behavior for deviated wells from naturally fractured-vuggy carbonate gas reservoirs. The study proposes a semi-analytical triple porosity model to determine the pressure and production behavior. The study determines that primary flow occurs only through fractures due to a relatively higher permeability contribution. The mathematical model was analyzed using Laplace transformation of the resulting dimensionless model of pseudo-pressure and pseudo-time. These were utilized to present point and line source solutions. The model was then bench-marked with field data from the Arum River Basin in Turkmenistan, confirming its validity. The model determines the pseudo pressure, which is then utilized to calculate the dimensionless flow rate. The gas flow rate is determined using the dimensionless groups provided in the study^16^.

5 $$\frac{1}{r}\frac{\partial }{\partial r}\left(r\frac{\partial {m}_{f}}{\partial r}\right)+\frac{{k}_{vf}}{{k}_{f}}\frac{{\partial }^{2}{m}_{f}}{\partial {z}^{2}}=\frac{1}{0.0864}\frac{{\phi }_{f}{\mu }_{g}{C}_{tf}}{{k}_{hf}}\frac{\partial {m}_{f}}{\partial {t}_{p}}-{\alpha }_{m}\frac{{k}_{m}}{{k}_{h}}\left({m}_{m}-{m}_{f}\right)-{\alpha }_{c}\frac{{k}_{c}}{{k}_{h}}({m}_{c}-{m}_{f})$$

The carbonate matrix comprises vugs, pores, and fractures, in which vugs are the dominant hydrocarbon and water storage, and fractures are the dominant contributor to the permeability. Wu et al.^25^ proposed a multiple-continuum conceptual model based on present geological data and results from the core sample for carbonate formations in China. Both single and multiphase transport behavior in this model is studied. The conceptual model proposed in this study considers fractured vuggy rock consisting of high permeable connected fractures, low permeable rock matrix, and various-sized vugs^26^. The study shed light on the complexity of the flow in a rock formation by indicating the occurrence of non-Darcy flow and other non-linear behavior. The non-Darcy flow is accounted for by utilizing the Forscheimer equation for correcting gas permeability. The line mass source is part of the source term, reflected in the transport model*.*

6 $$\frac{\partial }{\partial t}\left\{\phi \left({S}_{o}{\overline{\overline{\rho }}}_{dg}+{S}_{g}{\rho }_{g}\right)\right\}=-\nabla .\left({\overline{\rho }}_{dg}{v}_{o}+{\rho }_{g}{v}_{g}\right)+{q}_{g}$$

Li et al.^9^ proposed a dual-porosity binomial deliverability equation for tight carbonate reservoirs. The model was history-matched with certain multipliers for specific properties to match the field production data. The model was utilized in a reservoir simulator to study gas production from tight Carbonate. However, they focused on the macroscopic scale rather than the effects of pore radius on the complete production. The model incorporated physical rock properties, including rock compressibility and deformation under specific stress. With the incorporation of dual phase-gas and water, the model included the effect of water production on the cumulative gas production and rock deformation.

### Knudsen diffusion

Knudsen diffusion is usually found in porous nanopore media. Based on Knudsen's number, gas transport in porous media is segregated into four flow regimes—continuum flow regime, slip flow regime, transition regime, and free molecular regime. These flow regimes are depicted based on the flow conditions, including pore radii. Based on rock characterization done on tight Carbonate, 1–100 nm pore radii is the window of pore radii for the hypothetical model^6^. In congruence with the findings of Germanou et al.^27^, the proposed model will also include the effect of tortuosity, which is incorporated into the Knudsen diffusion coefficient (D_k_) in the apparent permeability model.

**Figure 1:**
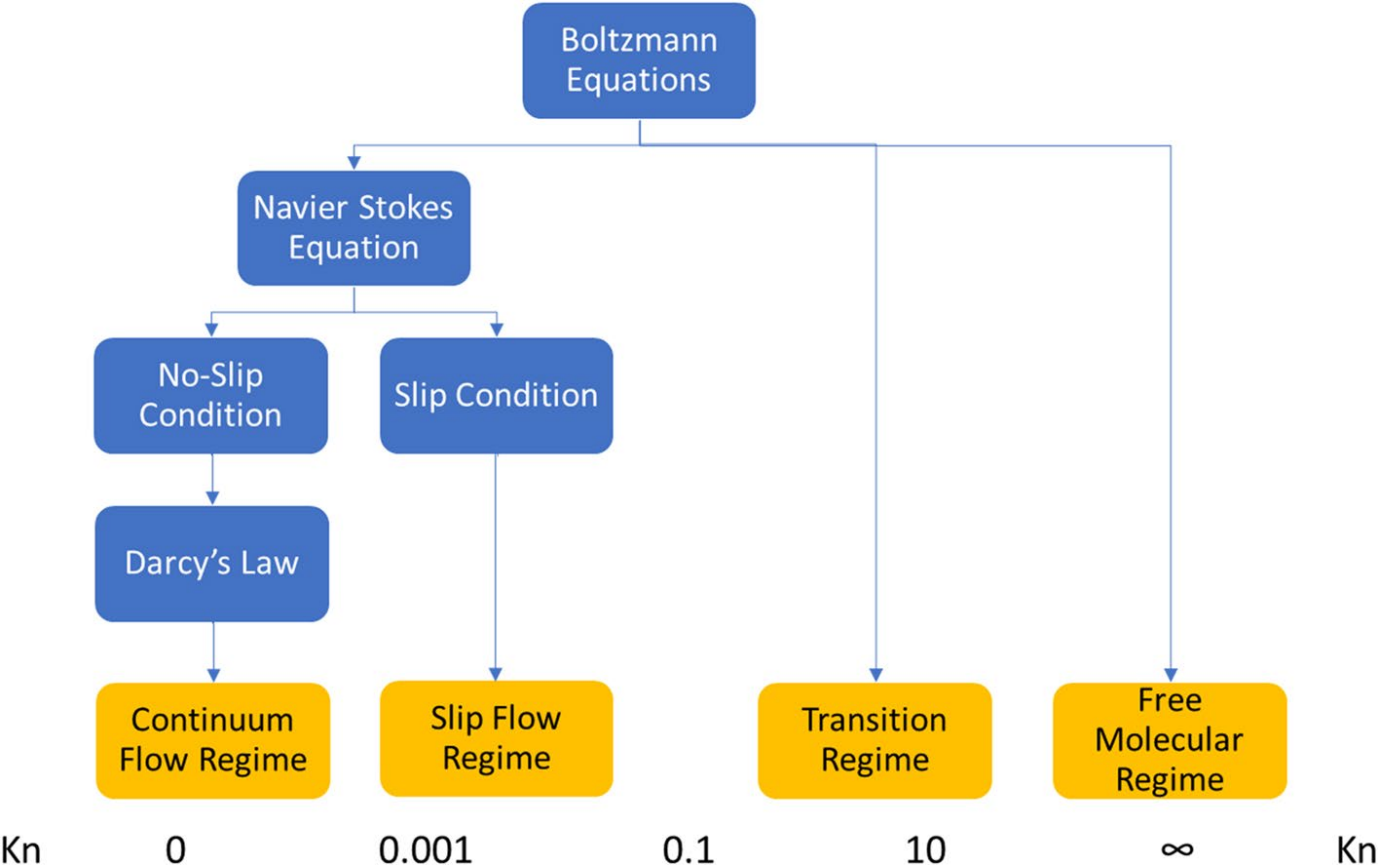
Flow regimes with corresponding Knudsen number. (Adapted from Jun et al.^30^).

**Figure 2:**
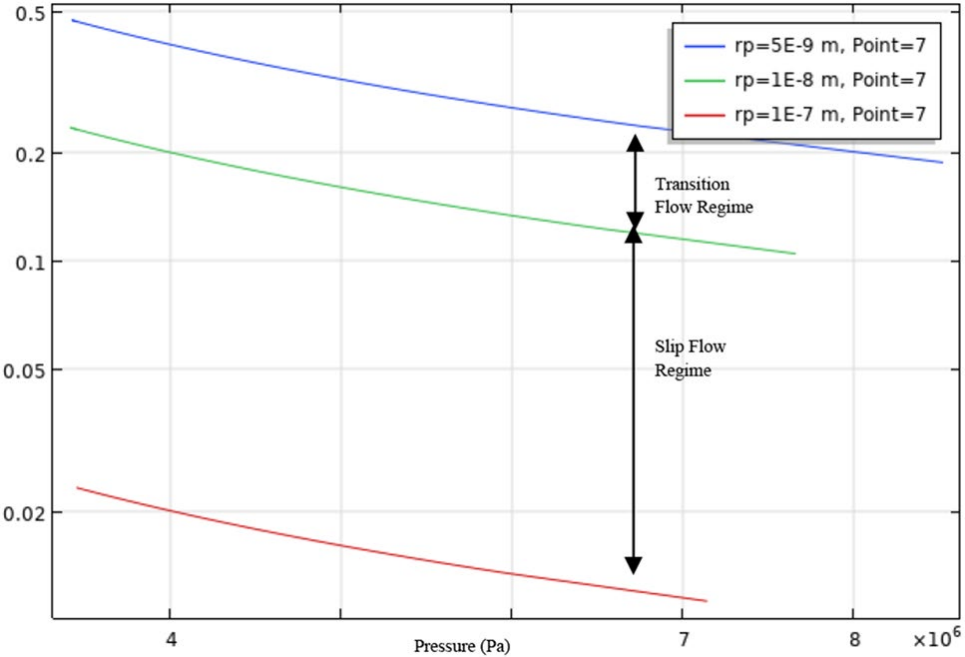
Knudsen Number based on pore radii and pressure of single-porosity model (rp = radius of the pore (m)).

Similar to transport in shale, carbonate reservoirs will undergo generally viscous flow and Knudsen diffusion due to the average pore throat size. Figures 1 and 2 show that the flow is not entirely a continuum, which confirms the non-Darcy flow occurring in the nanopores. It is crucial to include apparent permeability for gas transport to account for the gas pressure influence on the formation permeability. Various models effectively provide analytical formulations describing reservoir rocks' apparent permeability^28,29^.

### Model development

**Figure 3:**
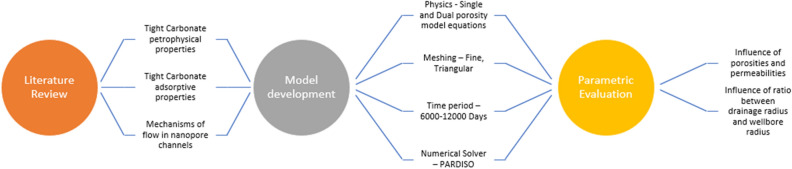
Methodology of study.

**Figure 4:**
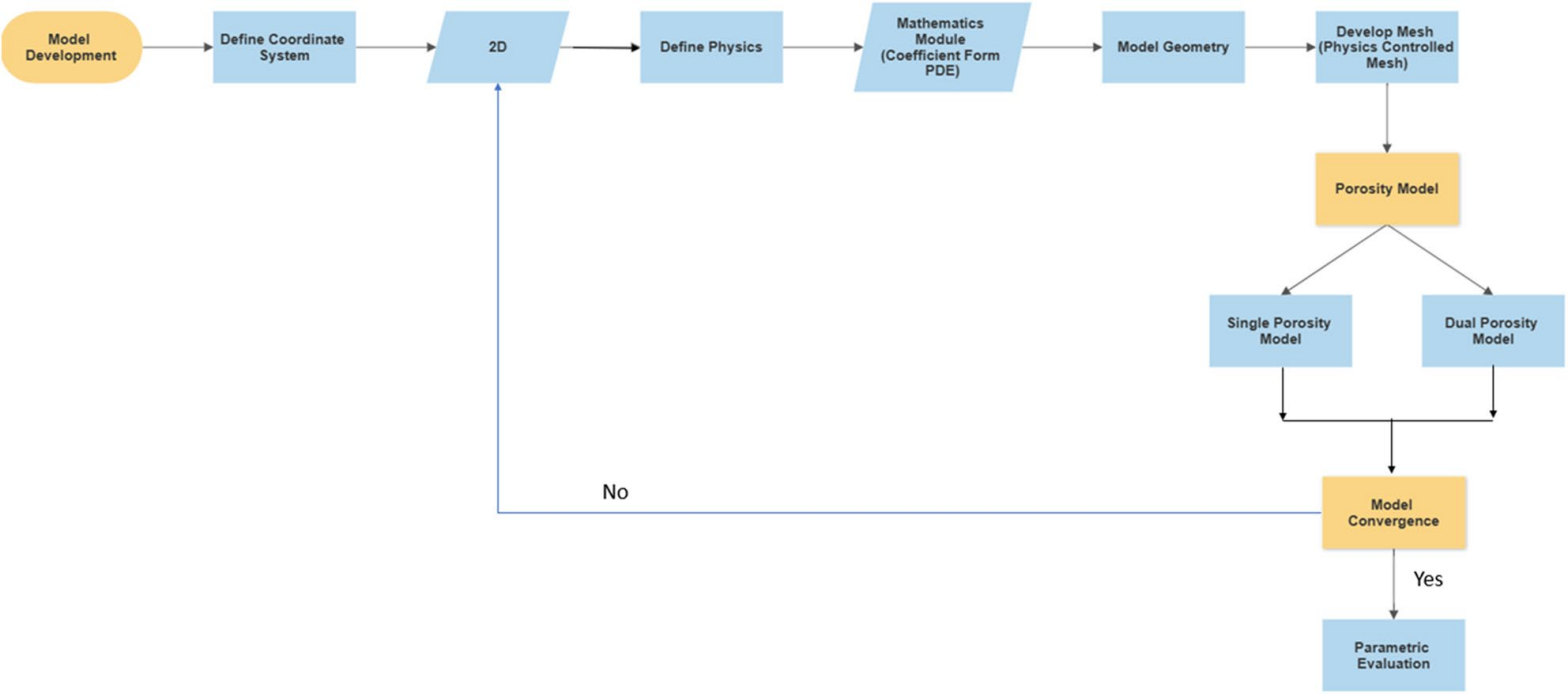
Model development methodology in COMSOL multiphysics.

This study develops the model in COMSOL Multiphysics v5.5 under 2D geometry. The 2D geometry assists in reducing the simulation time and considers thickness as 1 m or unity. 2D models were selected for this purpose due to the lack of field data. These models can assist in proving the concept through analytical solutions. The model is developed based on a specific workflow, as illustrated in Fig. 3. COMSOL Multiphysics was used to develop this model because it provides lumpsum and discretized analysis for any geometry. As the model is in partial differential form, the coefficient form PDE module is used to conduct the simulation for a specific period. Meshing utilized for the specific model was fine meshing with triangular discretization. The finite element method in COMSOL utilized a PARDISO solver for the numerical simulation under a period of 5000 to 12,000 days under different scenarios (Fig. 4).

The model is based on the following assumptions.

- The reservoir is assumed to be isothermal with gas adsorption on the matrix that can also fit the Langmuir isotherm equation.
- The mass continuity equation is considered.
- Single phase and component for fluid are only considered
- The homogenous matrix in terms of permeability in both the x and y direction
- A 2D model is developed with the reservoir thickness being 1m.

Based on findings from Rashid et al., there are multiple petrofacies found in tight carbonate reservoirs^6^. This study considers petrofacies containing only matrices and do not include effects from vuggy pores or fractures. As this is a more straightforward scenario, it also assists in providing a microscopic level of study with the inclusion of apparent permeability models and adsorption. Li et al. have done a similar analysis on tight carbonate reservoirs but have not included the desorption of natural gas^9^. Based on Elbashier et al.'s^11^ findings, there is an affinity towards CH_4,_ which can influence total gas production but is lower than CO_2_. For this specificity, the Langmuir isotherms of calcite are utilized to develop the reservoir model. Elbashier et al.^15^ concluded that geomechanical effects are negligible during any gas transport in tight Carbonate; therefore, no geomechanical coupling is required with the transport model in this particular study. However, utilizing apparent permeability is valid due to the adsorption of CH_4_ molecules on the calcite surface, as confirmed by Elbashier et al. and Onawole et al.^5,31^.

The Dusty gas model (Eq. 7) is used for this model as it incorporates the effects of Knudsen and ordinary diffusion and viscous flow, which is also described in the pore system of tight carbonate reservoirs (Li et al.^12^). The model is used to develop single and dual-porosity models using the following expression^12,30^.

7 $${k}_{a}={k}_{\infty }\left(1+\frac{{b}_{k}}{P}\right), {b}_{k}=\frac{{D}_{k}\mu }{{k}_{\infty }}$$

where ${D}_{k}$ is the Knudsen diffusion coefficient (m^2^/s) and ${b}_{k}$ refers to Klinkenberg Coefficient. The Model was also utilized similarly to the shale studies as it refers to flow in nanopores^32^.

#### Parameters for single-porosity model

The single-porosity model developed in this study incorporates only the tight matrix to contrast the stimulated reservoir and the tight reservoir; the model parameters are given in Table 1. Langmuir adsorption isotherm parameters are obtained from the literature that has conducted adsorption experiments on different tight carbonate reservoirs^5,31,33^.

**Table 1:** Parameters for single-porosity model.

Parameter	Value	Unit
Initial reservoir pressure	1.04 × 10^7^	Pa
Well bottom pressure	3.45 × 10^6^	Pa
Reservoir temperature	323	K
Matrix porosity	0.05	
Langmuir volume	0.233	m^3^/kg
Langmuir pressure	20	1/bar
Radius of pore	100	Nm
Wellbore radius	0.1	m
Drainage radius	121	m
Model dimensions	100 × 100	m
Matrix permeability	1 × 10^–20^	m^2^

#### Numerical model of single-porosity model

**Figure 5:**
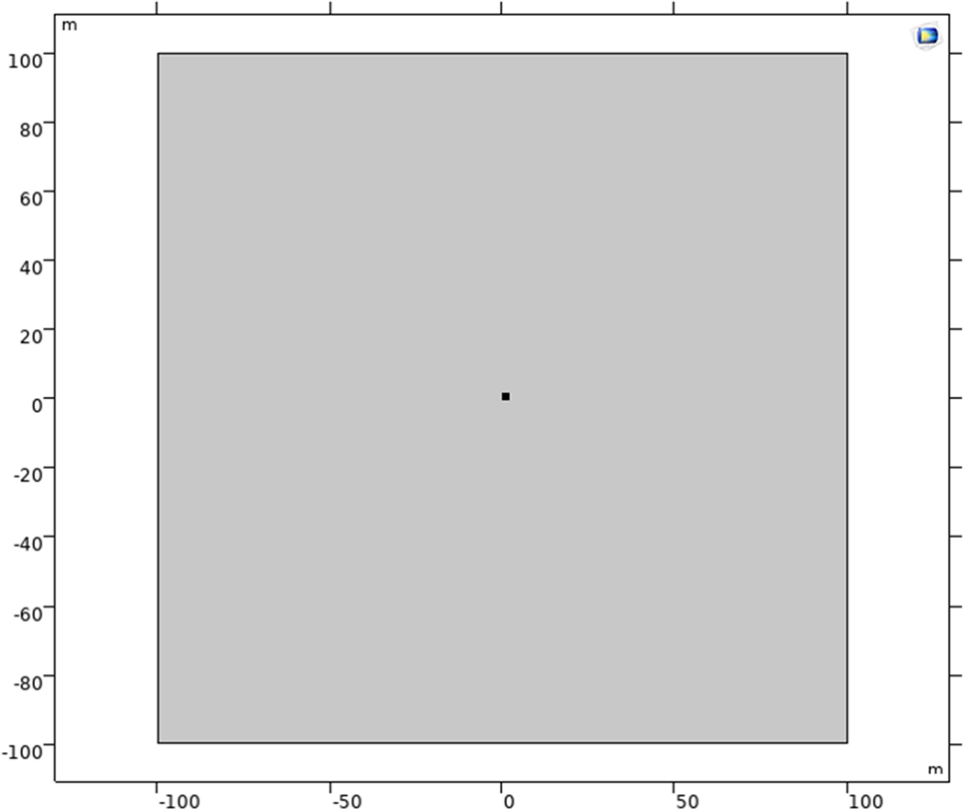
Model geometry for single-porosity model.

While using a single-porosity model (Fig. 5), properties of the matrix (Table 1) were utilized. These properties included a correction of the matrix permeability to gas apparent permeability (Eq. 7). Although the model utilized poor petrographic characteristics (porosity and permeability), the study on the formation was conducted to provide a base scenario and compare the influence of well stimulation in the following scenario. Contrary to the typical tight carbonate incorporating vugs and fractures, the model focuses on the matrix. Equation 8, determined to be a governing equation for the gas transport in tight formations, is considered for the single-porosity medium. The desorption term is also included in the governing equation representing the third flow mechanism apart from viscous flow and Knudsen diffusion.

8 $$\frac{d}{dt}\left({\rho }_{m}{\varnothing }_{m}\right)+ \nabla .{N}_{m}= -{Q}_{g}$$

where ${\rho }_{m}$ (kg/m^3^) is the density of gas present in the matrix, ${\varnothing }_{m}$ is the matrix porosity, ${q}_{ads}$ is the amount of adsorbed gas on the pore surfaces (Kg/m^3^), ${{\varvec{N}}}_{{\varvec{m}}}$ is the mass flux of gas through the porous media (kg/m^2^.s) and ${{\varvec{Q}}}_{{\varvec{g}}}$ is gas mass flow rate through the porous media (kg/s). The mass flow rate is expressed by Eq. 2^34,35^.

9 $${{\varvec{Q}}}_{{\varvec{g}}}= \frac{{\rho }_{m}{k}_{a}}{\mu }\frac{\theta }{\mathrm{ln}\left({r}_{e}-{r}_{w}\right)}({P}_{m}-{P}_{w})$$

where, ${r}_{e}$ refers to drainage radius (m), ${r}_{w}$ as wellbore radius, $\theta$ is the connecting factor which is valued at $\pi /2$ for well at the corner of the geometry, and $2\pi$ for well located at the center of the model as illustrated in Fig. 5, and ${P}_{w}$ is the wellbore pressure or bottom-hole pressure. As mentioned earlier, ${{\varvec{q}}}_{{\varvec{a}}{\varvec{d}}{\varvec{s}}}$ for tight carbonates is determined to be negligible.

#### Boundary conditions of single-porosity model

Initial conditions of the carbonate matrix are referred to ${P}_{i}$ (Pa), moreover, it covers the complete geometry of the model, which can be expressed as:

10 $${P}_{m}{|}_{t=0}= {P}_{i}$$

The outer boundary for the matrix is considered sealed, and the wellbore pressure determines the inner boundary (boundary of the wellbore).

11 $$\frac{\partial {P}_{m}}{\partial n} =0, {P}_{m}={P}_{w}$$

where n refers to the average unit vector of the model's boundary, represented as a Neumann boundary condition.

#### Parameters of dual-porosity model (matrix + acidized)

**Figure 6:**
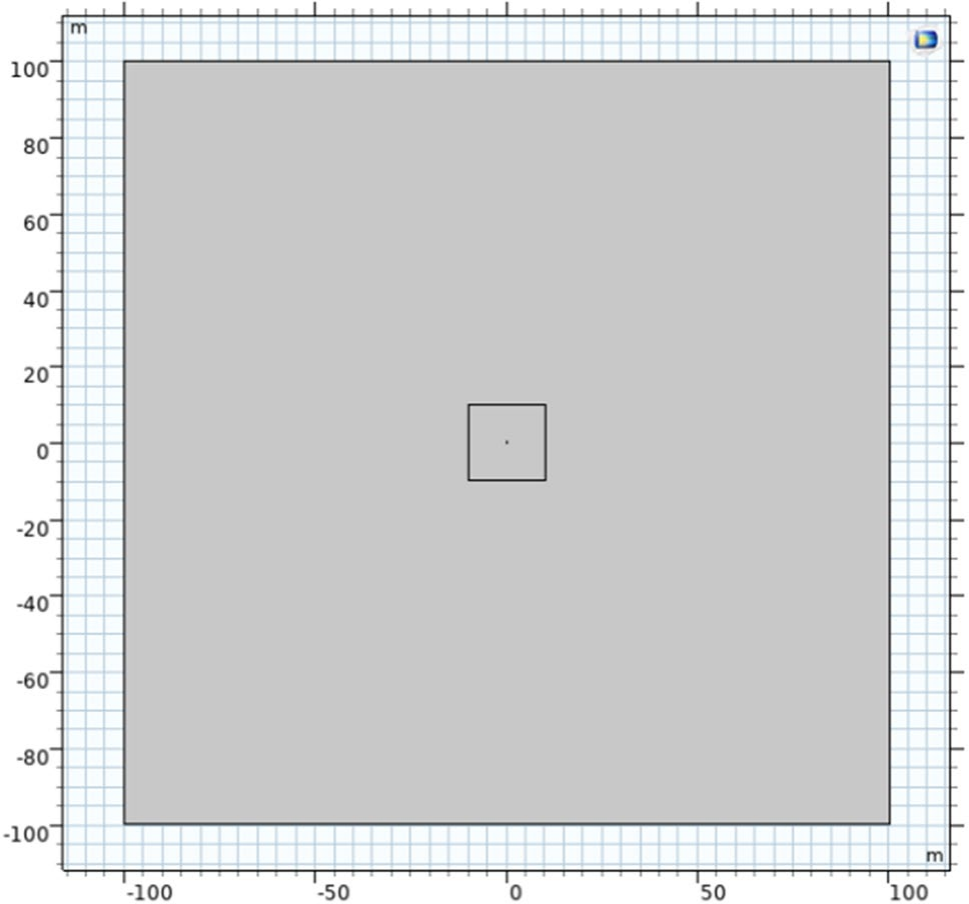
Model geometry for dual-porosity model.

The dual-porosity model (Fig. 6) focused on the stimulated reservoir (Acidization). The stimulated case included acidizing a small region around the well to increase the porosity and permeability. This approach would assist in increasing the total production of the reservoir. The model is known as dual-porosity due to the presence of different regions (acidized and non-acidized). Parameters associated with the dual-porosity model are shown in Table 2.

**Table 2:** Parameters for dual-porosity model.

Parameter	Value	Unit
Initial reservoir pressure	1 × 10^7^	Pa
Well bottom pressure	1 × 10^6^	Pa
Reservoir temperature	323	K
Matrix porosity	0.05	
Radius of pore	100	nm
Wellbore radius	0.1	m
Drainage radius	121	m
Fracture porosity	0.001	
Fracture permeability	1 × 10^–15^	m^2^
Reservoir dimensions	100 × 100	m

As mentioned earlier, certain assumptions govern the model to have a proper contrast with the single-porosity model, which include that gas is present in the nanopores as both adsorbed, and free, single component gas is present, and the model is homogenous and isothermal.

#### A numerical model for double porosity (matrix and acidized region)

Based on Yao et al.^30^ work on the double porosity model, and as the mathematical model is validated with an analytical solution, it is utilized to represent the two different regions. The reservoir model is a composite model due to two regions (acidized matrix and standard matrix). Each equation represents one domain in the geometry.

12 $$\left[\gamma {\varnothing }_{m}\right]\frac{{\partial P}_{m}}{\partial t}-\nabla .\left[\gamma \left[\frac{{P}_{m}{k}_{a,m}}{{\mu }_{m}}\left(\nabla {P}_{m}\right)\right]\right]=-\frac{\gamma {P}_{m}{k}_{a,m}}{{\mu }_{m}}\left({P}_{m}-{P}_{a}\right)$$

13 $$\left[\gamma {\varnothing }_{a}\right]\frac{{\partial P}_{a}}{\partial t}-\nabla .\left[\gamma \left[\frac{{P}_{a}{k}_{a,ac}}{{\mu }_{m}}\left(\nabla {P}_{m}\right)\right]\right]=\frac{\gamma {P}_{m}{k}_{a,m}}{{\mu }_{m}}\left({P}_{m}-{P}_{a}\right)-{Q}_{g}$$

The source term is referred to as *Q*_*g*_, which can be expressed as

14 $${{\varvec{Q}}}_{{\varvec{g}}}= \gamma \frac{{\rho }_{m}{k}_{a}}{\mu }\frac{\theta }{\mathrm{ln}\left({r}_{e}/{r}_{w}\right)}({P}_{a}-{P}_{w})$$

#### Numerical solution of dual-porosity model

The finite element method is utilized to solve the single and dual-porosity models proposed in this study. In the dual-porosity model, two dependent (P_a_ and P_m_) variables will be solved alternately. The pressure in the acidized region is solved initially, then the matrix pressure. Yao et al.^30^ provided a similar solution for the dual-porosity model, which can be utilized here. The following are the discrete-time domains for both variables by forward difference.

15 $$\gamma {\phi }_{a}\frac{{P}_{a}^{n+1}-{P}_{a}^{n}}{{t}^{n+1}-{t}^{n}}-\nabla .\left[\gamma \left[\frac{{P}_{a}^{n}{k}_{a}^{n}}{{\mu }_{a}^{n}}\left(\nabla {P}_{a}^{n+1}\right)\right]\right]={Q}_{p}^{n+1}-{Q}_{m}^{n+1}$$

16 $${Q}_{p}^{n+1}= \frac{\gamma {P}_{m}^{n}{k}_{m}^{n}}{{\mu }_{m}^{n}}\left({P}_{m}^{n}-{P}_{a}^{n}\right)$$

17 $${Q}_{m}^{n+1}= \gamma \frac{{P}_{a}^{n}{k}_{a}^{n}}{{\mu }_{a}^{n}}\frac{\theta }{\mathrm{ln}(\frac{{r}_{e}}{{r}_{w}})}({P}_{a}^{n}-{P}_{w})$$

#### Boundary conditions of dual-porosity model

The initial pressure of the matrix and acidized region is depicted as ${P}_{i}$. Therefore, the initial conditions of the reservoir are:

18 $${P}_{m}{|}_{t=0}={P}_{a}{|}_{t=0}= {P}_{i}$$

The outer boundary condition has no slip and is a closed boundary, while the inner boundary refers to the wellbore. The outer boundary condition can be expressed as

19 $$\frac{\partial {P}_{a}}{\partial n} =\frac{\partial {P}_{m}}{\partial n}|{r}_{1}=0,$$

while the inner boundary condition is

20 $${P}_{a}|{r}_{2}={P}_{w}$$

## Discussion and results

### Model validation

**Figure 7:**
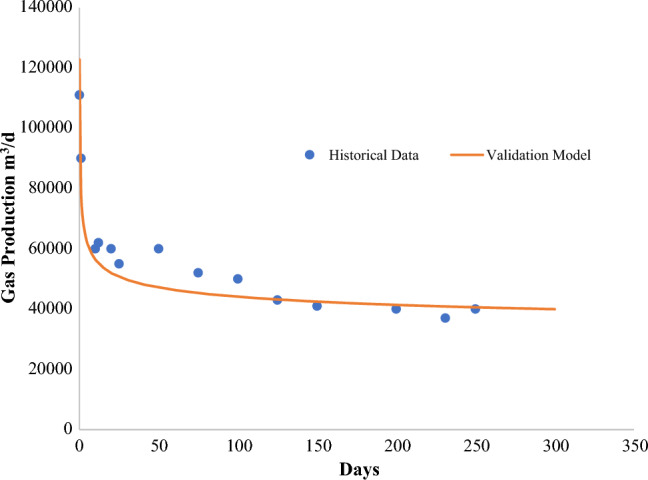
Historical data of marcellus shale and validation model.

Due to lack of field data for tight carbonate reservoirs, the model is validated using field data from Marcellus shale gas production (Fig. 7). Due to its availability, numerous researchers use this data as a standard, and it can also be represented by different shale models. The parameters needed to validate the gas transport model are listed in Table 3. Due to a little inaccuracy in the manufacturing profiles, geomechanical influence is disregarded in comparison to Micheal et al.^36^, who used a similar validation process. With a maximum inaccuracy of up to 9%, Fig. 4 shows that the gas transport model agrees well with the real-field data. This is to be expected given the model's assumptions.

**Table 3:** Reservoir parameters for validation model.

Parameters	Marcellus Shale	Units
Reservoir dimension	1219.2 $\times$ 304.8 $\times$ 52.7	m
Langmuir pressure	3 $\times$ 10^6^	Pa
Langmuir volume	2.5 $\times$ 10^–3^	m^3^/kg
Initial reservoir pressure	34.5 $\times$ 10^6^	Pa
Bottomhole pressure	2.4 $\times$ 10^6^	Pa
Reservoir temperature	352	K
Gas viscosity	2 $\times$ 10^–5^	m.s
Hydraulic fracture width	0.003	–
Initial fracture permeability	30	mD
Hydraulic fracture spacing	30.5	m
Hydraulic fracture half-length	85.3	m
Number of fractures	14	–
Initial matrix permeability	1 $\times$ 10^–19^	m^2^
Initial fracture porosity	0.03	–
Possion’s ratio	0.2	–

The validation model and Marcellus Shale historical data show a good degree of agreement. In 250 days, the gas production rate drops from 9 × 10^4^ m^3^/d to 3.7 × 10^4^ m^3^/d. Cao et al. had a good agreement and also used historical data from Marcellus shale. The validation model has inconsistencies because the adsorption properties did not appear to have a significant influence on it^37^. To specify various parameters for a subsequent parametric study using DCA to validate it, another parametric investigation was carried out. According to other studies^36,38^ Langmuir Volume was found to have very little effect on the total production.

### Mesh sensitivity analysis

**Figure 8:**
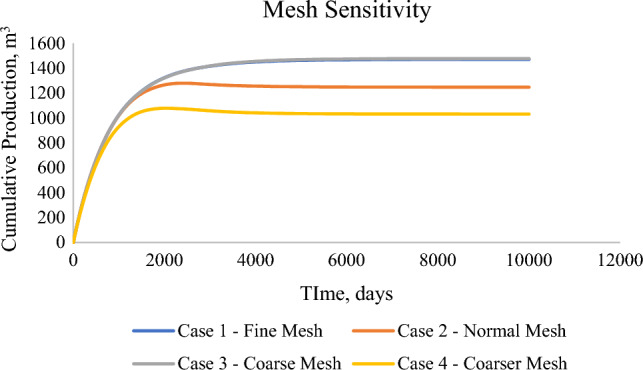
Cumulative production with varying mesh size.

The mesh sensitivity analysis was conducted for the single porosity model with the production flowrate as the parameter. Based on the in-built option of parametric studies, the sensitivity analysis was conducted on the following criteria that defines the particular mesh sizing—Maximum element size, minimum element size, maximum growth rate, resolution of curvature, and resolution of narrow regions. Table 4 provides an overview of the cases utilized. The results show that with an increase in maximum element size, the results deviate from the original results, with a decrease in computational time. Case 1 and Case 3 as per Fig. 8—Cumulative production with varying mesh size provided conclusive results, while during case 2 and case 4, the model stops converging after 3000–4000 days, so the cumulative production is very low. Due to such results, fine mesh (case 1) is utilized. Complete parametric analysis was completed in 14.8 min with the computational machine of Intel ® Xeon® Gold 6226R CPU (2.9 GHz) and RAM of 192 GB.

**Table 4:** Sensitivity analysis—case details.

Cases	In-built type	Maximum element size	Minimum element size	Maximum element growth rate	Curvature factor	Resolution of narrow regions
Case 1	Fine	10.6	0.06	1.3	20	1
Case 2	Normal	13.4	0.06	1.3	0.4	1
Case 3	Coarse	20	0.4	1.4	1.4	1
Case 4	Coarser	66	10	2	2	0.9

### Production profile of single-porosity

**Figure 9:**
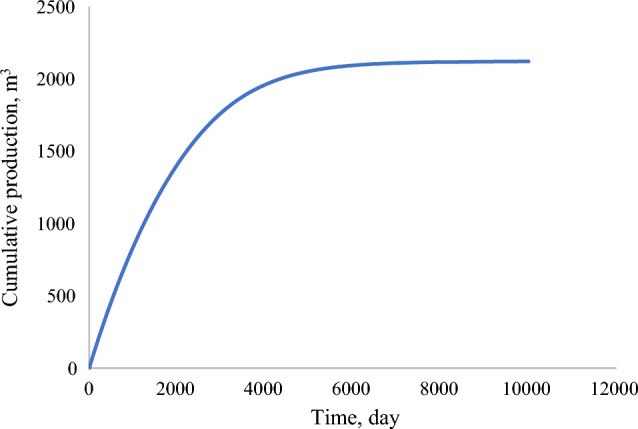
Cumulative Production (m^3^) from single-porosity model.

The model in 2D geometry had an exceptionally low production, which capped at 2119 m^3^ in 6000 days, which can be drawn back to having a 0.33 m^3^/d average production rate. Figure 9 depicts the cumulative production occurring in 12,000 Days. It can be observed that with just a production gradient, there is very low production and recovery from these reservoirs. Therefore, adding natural fractures or well-stimulation can effectively enhance the recovery from the reservoir.

### Production profile of dual-porosity model

**Figure 10:**
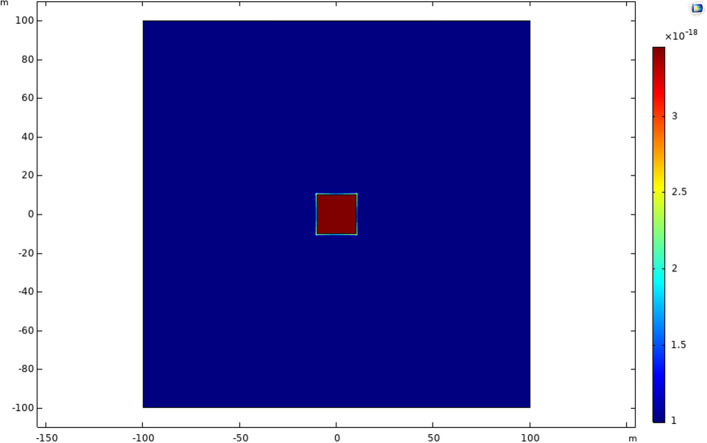
Formation permeability at day 0.

**Figure 11:**
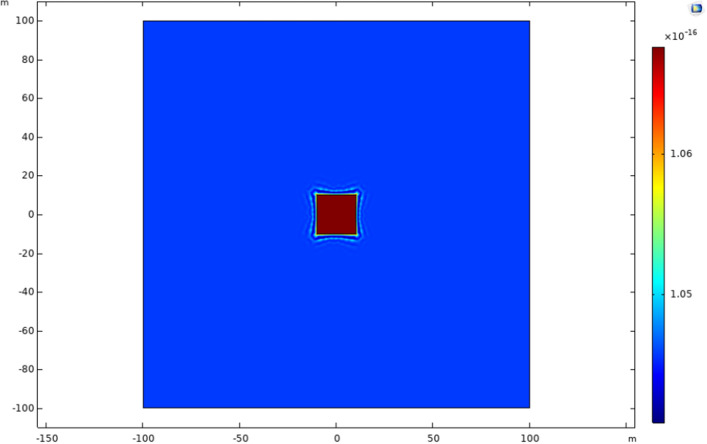
Formation permeability at day 5000.

The dual-porosity model comprised three domains—the matrix region, the acidized region, and the wellbore. The matrix region represents the tight formation with low porosity and permeability, while the acidized region depicts the formation with enhanced transport properties due to well-stimulation techniques. In contrast to the matrix, the acidized domain has higher permeability, as depicted in Figs. 10 and 11. When the apparent permeability is introduced into the model, the apparent permeability of the matrix increases with the decrease in matrix pressure. However, this occurrence was not observed in the acidized region due to higher permeability for the formation and evidence provided in studies by Yao et al.^30^.

**Figure 12:**
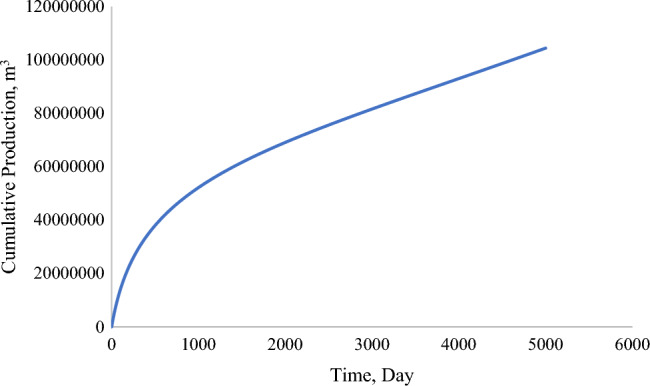
Cumulative production (m^3^) from stimulated well (dual-porosity model).

**Figure 13:**
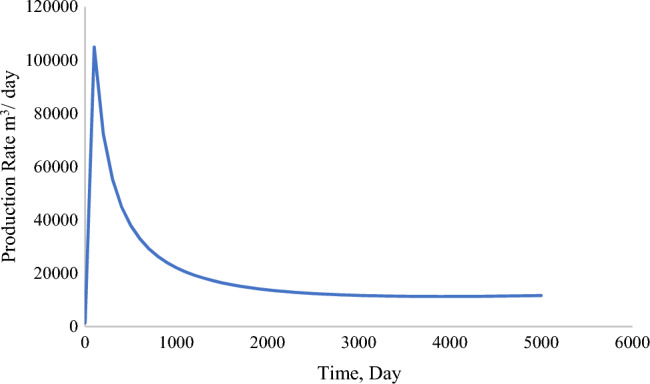
Production rate (m^3^/Day) from stimulated well (dual-porosity model).

Production from the acidized reservoir increased substantially in contrast to the single-porosity model. The total production peaked at 1.2 × 10^8^ m^3^ by day 5000 and at 104,901 m^3^ per Day (Figs. 12, 13). This contrast is attributed to various factors, which will be discussed further in the parametric evaluation of the dual-porosity model.

### Parametric evaluation

Parametric evaluation is conducted on the acidized model based on permeability and the drainage and wellbore radius ratio. This analysis is performed to determine the changes in the total production caused by the physical properties of the formation and wellbore configuration.

#### Porosity and permeability

**Figure 14:**
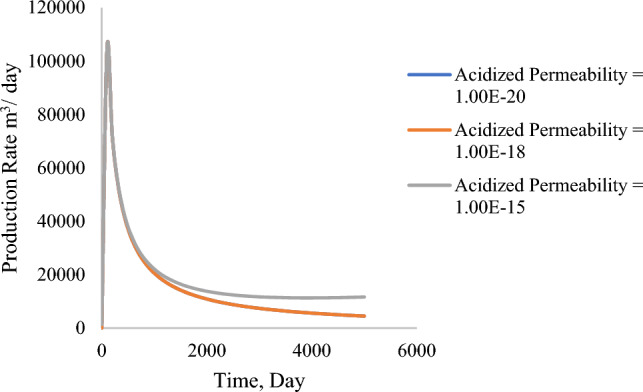
Production profile based on the change in acidized formation permeability (m^3^).

**Figure 15:**
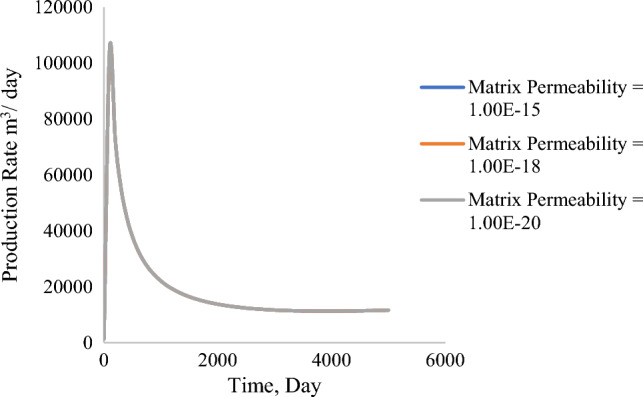
Production profile based on tight matrix permeability (m^3^).

Increased permeability increases the degradation time of the production, and the production rate remains the same. In theory, this can be due to increased space for gas flow toward the wellbore. Figures 14 and 15 illustrate the variation of both acidized and matrix permeability. The matrix permeability was determined to have negligible influence on the production rate from the formation, whereas the acidized permeability adversely influences gas production. With lower acidized region permeability, there was an increase in total production by 8%.

#### The ratio between the radius of drainage and the radius of the wellbore (r_e_/r_w_)

**Figure 16:**
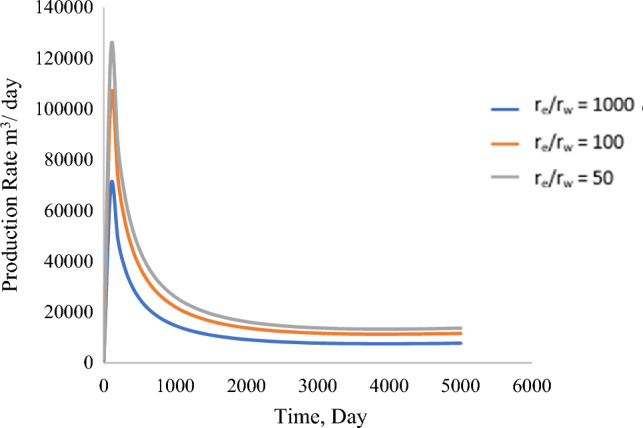
Production profile based on the change in the ratio of drainage radius and wellbore radius (r_e_/r_w_).

Determining the influence of wellbore radius and drainage radius is vital for any production analysis. Based on the findings from Fig. 16, it is concluded that total production decreases as the drainage radius increases. The model was simulated under boundary conditions that portray pressure conditions at the wellbore and reservoir; these results are valid as they represent faster production with more wellbore radius.

## Conclusion

The study implemented a single and dual-porosity transport model to estimate the gas production in tight carbonate reservoirs while including the effects of sorptive properties of the formation. The study initially presented similarities between the tight shale formation and tight carbonate formation with its presence of nanopores and the dominance of the nanopores onto the porosity, while microfractures dominate over permeability. The following points are the summarization of all the key findings.

- Tight carbonate reservoirs are similar to other tight gas reservoirs; the only distinguishing aspect is the negligible methane adsorption occurring in calcite. The findings were based on the molecular simulations and adsorption experimentations done under the same research project.
- Two scenarios were studied in which single and dual-porosity models are developed and utilized. The proposed scenarios included effects of Knudsen diffusion and viscous flow through the gas apparent permeability model and desorption through the governing equation.
- The single-porosity model was developed to describe the difficulty in production from the nanopore matrix directly. Regardless of pressure drawdown from the reservoir to the wellbore, the model depicted a very low production rate, which is not feasible on all accounts.
- It was observed during model development that the proposed model is only suitable for formations with pore radii between 1 and 100 nm, corresponding to implementing flow mechanisms.
- The models are prepared in 2D due to the lack of field data for validation.

For future research, the proposed model must be effectively modified depending on the various petrofacies, such as including a triple porosity model that describes vuggy-matrix and fractures, even though the model was validated by analytical solutions published in the literature. It is possible through experimental simulation on tight carbonate core samples. The proposed models lay a foundation for a tight carbonate transport model for gas.

## Data Availability

The data used in this study will be available for readers upon request. Please contact Prof. Ibnelwaleed Hussein (ihussein@qu.edu.qa) in this regard.
