# Author Correction: CFD estimation of gas production in tight carbonates using single and dual-porosity models

**DOI:** 10.1038/s41598-024-53553-8

**Published:** 2024-02-05

**Authors:** Syed Oubee Khadri, Ibnelwaleed A. Hussein, Fadhil Sadooni, Ezeddin Shirif

**Affiliations:** 1 Gas Processing Center, Qatar University, P.O. Box 2713, Doha, Qatar; 2 Department of Chemical Engineering, Qatar University, P.O. Box 2713, Doha, Qatar; 3 Environmental Science Center, Qatar University, P. O. Box 2713, Doha, Qatar; 4 Program of Petroleum Systems Engineering, University of Regina, Regina, SK S4S 0A2 Canada

Correction to: *Scientific Reports* 10.1038/s41598-023-48450-5, published online 19 December 2023

The Funding section in the original version of this Article was incorrect.

"Qatar National Research Fund, NPRP11S-1228-170138 and NPRP12S-0305-190235, NPRP11S-1228-170138 and NPRP12S-0305-190235, NPRP11S-1228-170138 and NPRP12S-0305-190235."

now reads:

“Qatar National Research Fund, NPRP11S-1228-170138, NPRP12S-0305-190235, NPRP12S-013-190023 and NPRP13S-1231-190009.”

The original Article has been corrected.

